# Trends of rhinoplasty research in the last decade with bibliometric analysis

**DOI:** 10.3389/fsurg.2022.1067934

**Published:** 2023-01-06

**Authors:** Xuanru Zhu, Bin Zhang, Yuesheng Huang

**Affiliations:** ^1^ Department of Wound Repair, Southern University of Science and Technology Hospital, Institute of Wound Repair and Regeneration Medicine, Southern University of Science and Technology School of Medicine, Shenzhen, China; ^2^ Department of Burn and Plastic Surgery, General Hospital of Southern Theater Command of PLA, Guangzhou, China

**Keywords:** rhinoplasty, bibliometric analysis, hotspots, 3D anthropometry, outcome evaluation

## Abstract

**Background:**

As rhinoplasty (RP) with different requirements is becoming more and more popular in the latest decade, this study aims to quantitatively and qualitatively explore the trends in RP research, depict research hotspots, and point out the future direction with a bibliometric analysis.

**Methods:**

All RP literature studies in the last decade (from 2012 to 2021) were retrieved from the Web of Science Core database. Annual output, institutions, authors, journals, and most-cited literature studies were analyzed by bibliometric tools, including CiteSpace, bibliometric online platform, bibliometrix R language kit, BICOMB, and gCLUTO.

**Results:**

A total of 2,590 RP research studies dated between 2012 and 2021 were included according to our criterion. As for the country, the United States, Turkey, and Korea maintained the top three in RP research. As for the institutions, the University of California, Irvine, Stanford University, and University of Ulsan ranked top three in RP research publications based on article counts. Professor Rhorich RJ, Most SP, and Jang YJ were the most contributed authors according to article counts and citation number. The top journals were *The Plastic and Reconstructive Surgery, JAMA Facial Plastic Surgery*, and *Aesthetic Surgery Journal*. The 10 most-cited literature studies were also listed explicitly in this study. Finally, biclustering analysis on the most frequent keywords were conducted which helped us to identify seven hotspot clusters in RP research.

**Conclusions:**

We comprehensively summarized the publication information of RP literature studies in the past decade, highlighted the current status and trends over time, and provide guidance for in-depth research direction on RP for the future.

## Introduction

The nose is considered the most noteworthy feature in the face. Rhinoplasty (RP) is one of the most popular surgical cosmetic procedures performed worldwide, which improves the appearance or/and function, within the domain of the plastic surgeon as well as the otorhinolaryngologist (1). Ever since the first thesis that illustrated nose surgery by Edwin Smith Papyrus in 1930 (2), literature studies concerning RP are increasing with the rapid growth of surgical procedures being performed, including new methods, basic research, outcome measurement, artificial intelligence, and so on. However, it is still an inspiring task to imply macroanalysis based on a large amount of literature data to grasp the development trend of a particular field accurately, especially in such an extremely intricate field as RP.

In recent years, bibliometric analysis has gained great attention for it can evaluate quality trends through literature metrology. Meanwhile, research trends or hotspots within a certain field can be predicted. However, very few bibliometric studies on RP are available now. Only Sinha et al. summarized the 100 most cited articles in RP (3) and Lalezari et al. evaluated global trends in rhinoplasty research spanning 20 years between 1994 and 2013 (4). The former lacks a fully and comprehensively analysis due to the limited scope of included literature. The latter lacks updated information in the latest decade. Both of them mainly focus on publication information instead of analysis and prediction of research hotspots. Therefore, research hotspots verified through co-occurrence keywords biclustering in the last decade were highlighted in this study (5), providing a reference for in-depth research direction and clinical practice related to RP.

## Materials and methods

### Data sources and search strategy

All literature studies are searched and downloaded from the Web of Science (WOS) core collection. Search phrases are as follows: topic = (rhinoplasty) AND publication date = (January 1, 2012–December 31, 2021)) AND language = (English) AND document type = (Article, Review). All data were extracted in one day (December 31, 2021).

### Data recording

Two independent reviewers (XZ and BZ) both recorded the original data and conducted the primary search, with a coincidence rate of 95% or more. Any differences were raised and discussed until the reviewers reach a consensus.

### Publication analysis

Publishing characteristics including countries, institutions, authors, journals, and most-cited articles are presented in this analysis, which were all carried out by a bibliometric online platform (https://bibliometrix.org/).

### Network map presentation

CiteSpace is an optimal tool to depict collaboration network according to different publication characteristics. The principle of the CiteSpace operation is based on co-citation analysis and pathfinder network multidimensional scaling (6, 7). In this article, particular aspects, including connections and influence among institutions, authors, and co-cited authors are being vividly represented by CiteSpace network analysis. Centrality is the most representative indicators to value the importance of nodes in the network. Typically, higher centrality means the greater importance of the node among the whole network. Meanwhile, high citation keywords, which are also called burst words, within years are revealed to illustrate the research frontiers and focal point.

### Thematic map illustration

The thematic map was illustrated by bibliometrix R language kit; the four quadrants drawn represent the following:

The first quadrant (upper right corner): motor themes; it is an important and well-developed theme. The second quadrant (upper left corner): highly developed and isolated themes; it has developed well but is not important for the current field. The third quadrant (lower left corner): degrading themes and marginal themes, which have no good development and may just emerge or disappear. The fourth quadrant (lower right corner): basic and transitional themes; generally, it refers to basic concepts which are important in the field, but have not been well developed.

### Hotspots detection and clustering

BICOMB (Bibliographic Item Co-Occurrence Matrix Builder) is a software used to construct a co-occurrence keyword binary matrix that reveals the connections between the extremely frequent keywords. Once the matrix is built, we then imported the matrix into software gCLUTO (5, 8) and set appropriate parameters to get a matrix visualization as well as mountain visualization, which represented the semantic relationship between keywords and source literature studies. All mountain image features including colors, plane, altitude, peak, and volume are the reflection of associated clusters. The volume of each peak is directly proportional to the number of keywords appeared in this category, and the altitude of each peak shows the positively correlation of keywords within the same category. Also, the closer the peaks are, the more similar the clusters they represent. The internal standard deviation of keywords is revealed by the color of each peak. Red indicates a low deviation, while blue indicates the high one. The matrix values are represented graphically by colors, The color of each reseau paints the proportional emergence frequency of a major keywords in a literature. The colors gradually deepen from white to red, indicating that the keywords are less important to more important. Finally, the framework of RP research hotspots was generated based on the above visualization, and further studies were carried out according to representative papers in each cluster.

## Results

### The output of literature studies

**Figure 1:**
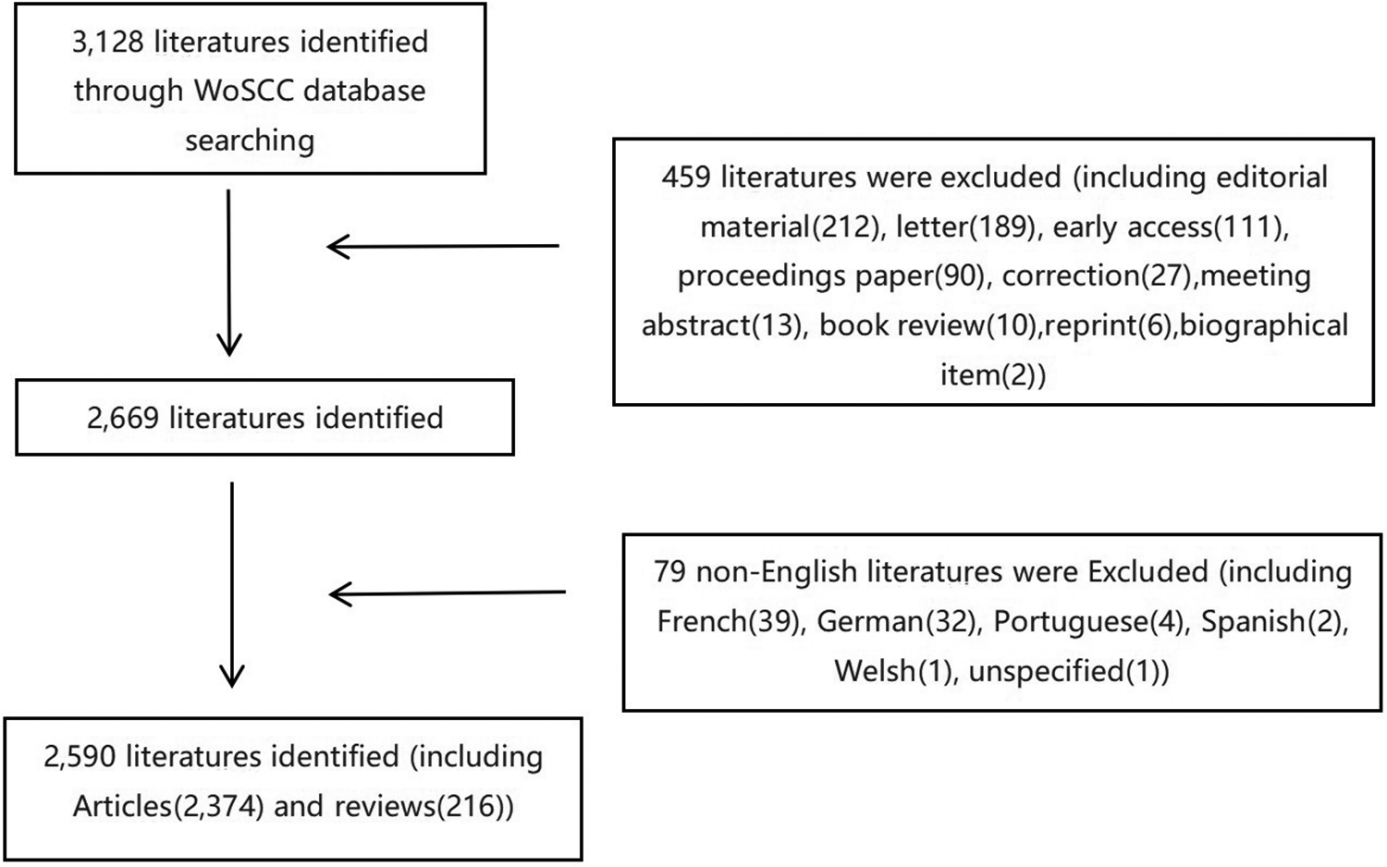
Flow chart of literature filtering in this study. WoSCC, Web of Science Core Collection.

**Figure 2:**
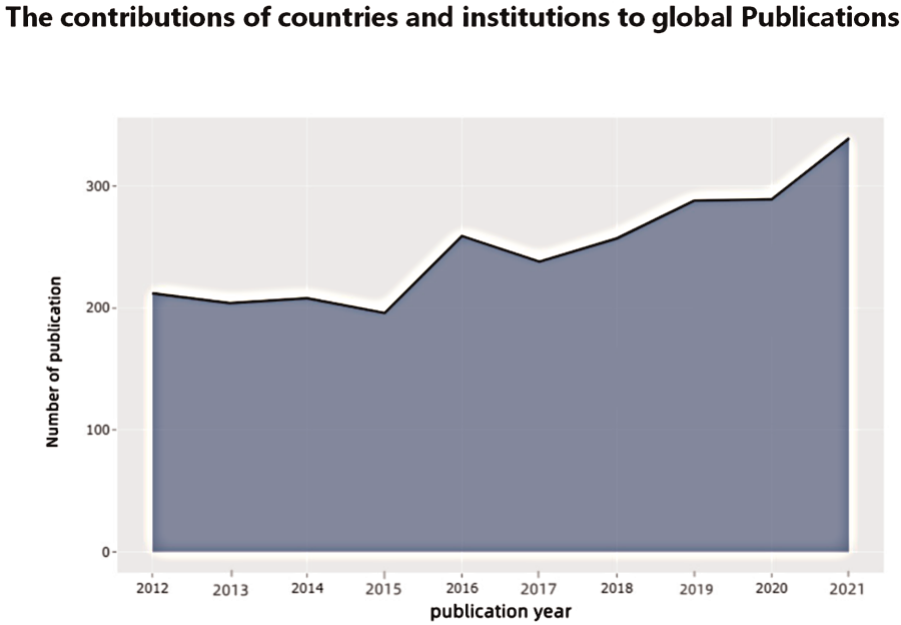
The annual number of publications worldwide.

From 2012 to 2021, a total of 3,128 publications were retrieved, according to our inclusion criteria, 538 publications were excluded, and 2,590 publications were used for bibliometric analysis; among them, 2,374 are articles and 216 are reviews (Figure 1). Figure 2 shows the successively increasing trend in the numbers of RP-related publications.

### The top 10 contributed countries and institutions worldwide

**Figure 3:**
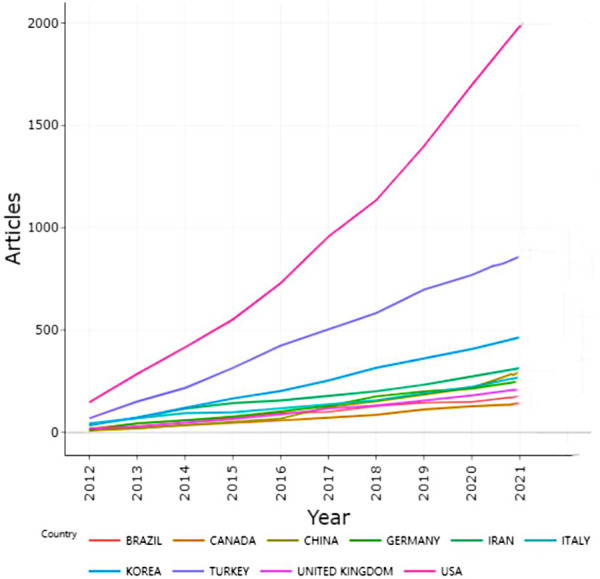
Publication status of the top 10 countries in RP research from 2012 to 2021.

The incorporated literature studies on RP were contributed by at least 84 different countries or regions (Figure 2). The United States (810, 31.27%) was the largest contributor to RP research, followed by Turkey (384, 14.83%), Korea (235, 9.07%), China (146, 5.64%), and Iran (112, 4.32%) (Figure 3). Thus, the results showed that the United States had more impact than any other country (centrality = 0.66), followed by Germany (0.12) and England (0.11). In terms of research institutions, the top five included the University of California, Irvine (130, 5.02%), Tehran University of Medical Sciences (119, 4.59%), Stanford University (114, 4.40%), University of Ulsan (106, 4.09%), and University of Pennsylvania (69, 2.66%) (Table 1).

**Table 1:** The top 10 countries/regions and institutions contributing to publications in rhinoplasty research.

Rank	Country	Article counts	Percentage	Centrality	Institutions	Article counts	Centrality	Total number of citation	Average number of citation
1	Unite States	810	31.27	0.66	Univ Calif Irvine	103	0.04	800	7.77
2	Turkey	384	14.83	0.04	Univ Tehran Med Sci	86	0.11	301	3.50
3	Korean	235	9.07	0.08	Stanford Univ	76	0.09	422	5.55
4	China	146	5.64	0.01	Univ Ulsan	58	0.09	321	5.53
5	Iran	112	4.32	0.01	Univ Penn	51	0.13	119	2.33
6	Germany	105	4.05	0.12	Harvard Med Sch	45	0.12	110	2.44
7	Italy	105	4.05	0.08	Univ Toronto	40	0.07	108	2.70
8	United Kingdom	79	3.05	0.11	Seoul Natl Univ	36	0.04	238	6.61
9	Brazil	63	2.43	0.05	Shanghai JiaoTong Univ	35	0.03	34	0.97
10	Canada	58	2.24	0.00	Univ Illinois	32	0.07	221	6.91

**Figure 4:**
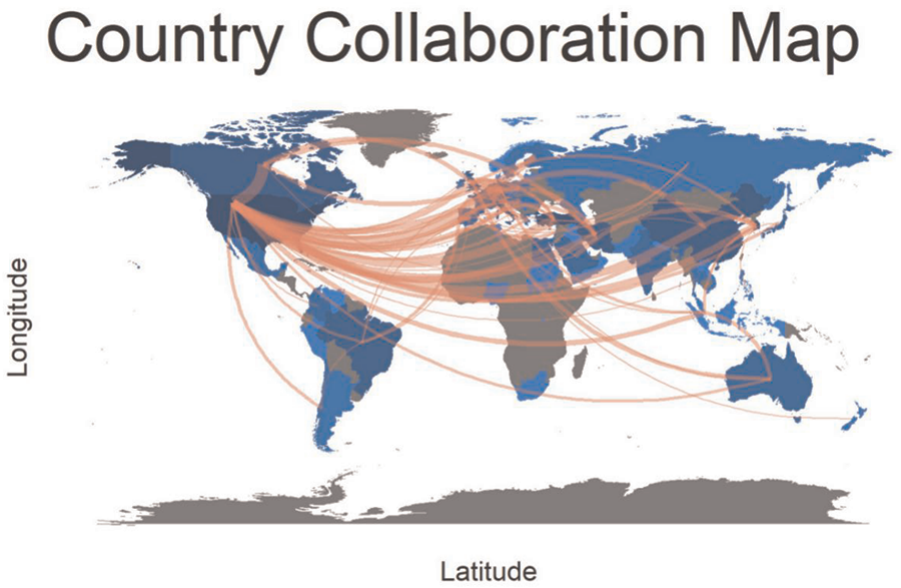
The worldwide country collaborative map in the field of RP.

**Figure 5:**
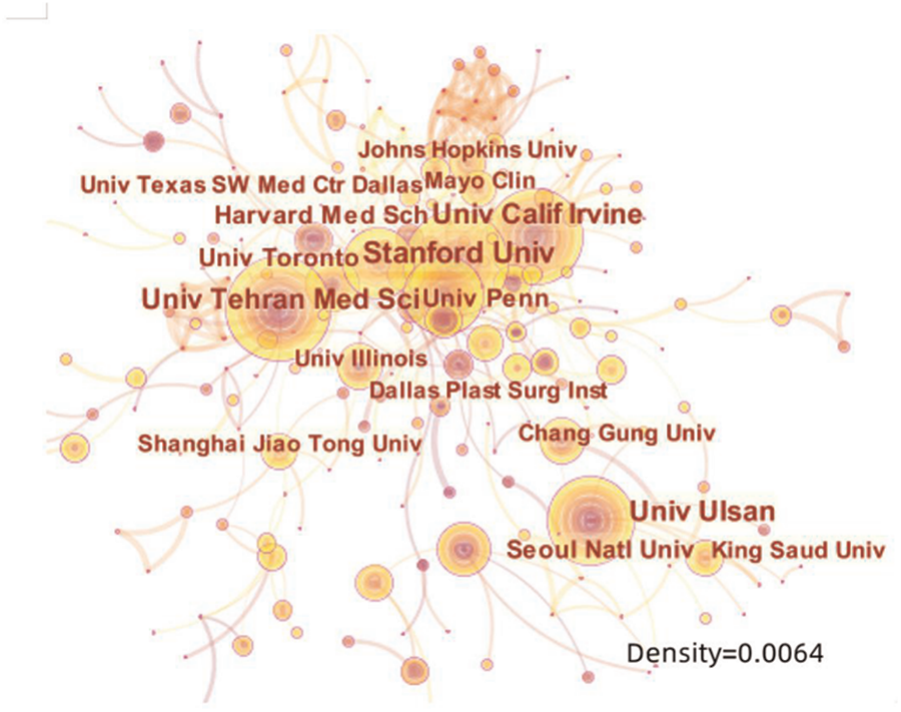
The network map of institutions that involved in rhinoplasty research (density = 0.0064).

The international cooperation map reveals that the United States cooperates most frequently with other countries, especially with Canada, Iran, and Germany. Turkey ranks the second according to publication number; however, it cooperates much less frequently with other countries (Figure 4). A low-density (density = 0.0064) map of the rhinoplasty research network (Figure 5) represents that the research groups were sparsely distributed in various institutions worldwide, thus communications and co-operations need to be intensified. For 7 out of 10 institutions, the central indexes are below 0.1, indicating that most institutions had a relatively low level of influence worldwide and did not cooperate closely enough in the recent 10 years. While the output of University of Pennsylvania was not very high, it had the greatest influence (0.13) among the RP region.

### The top 10 prolific authors on RP research

**Figure 6:**
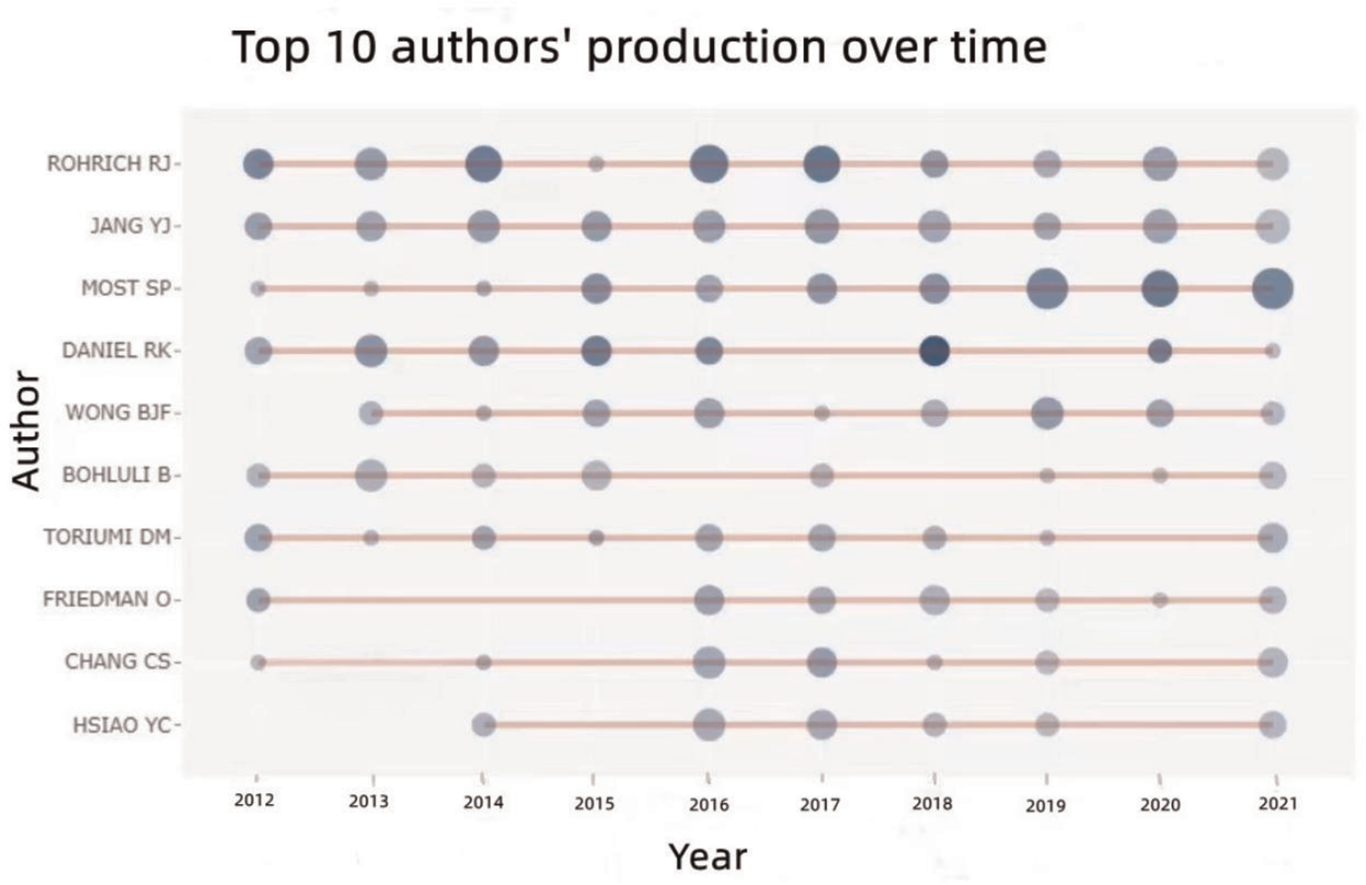
The annual output of literature studies by the top 10 authors from 2012 to 2021.

**Figure 7:**
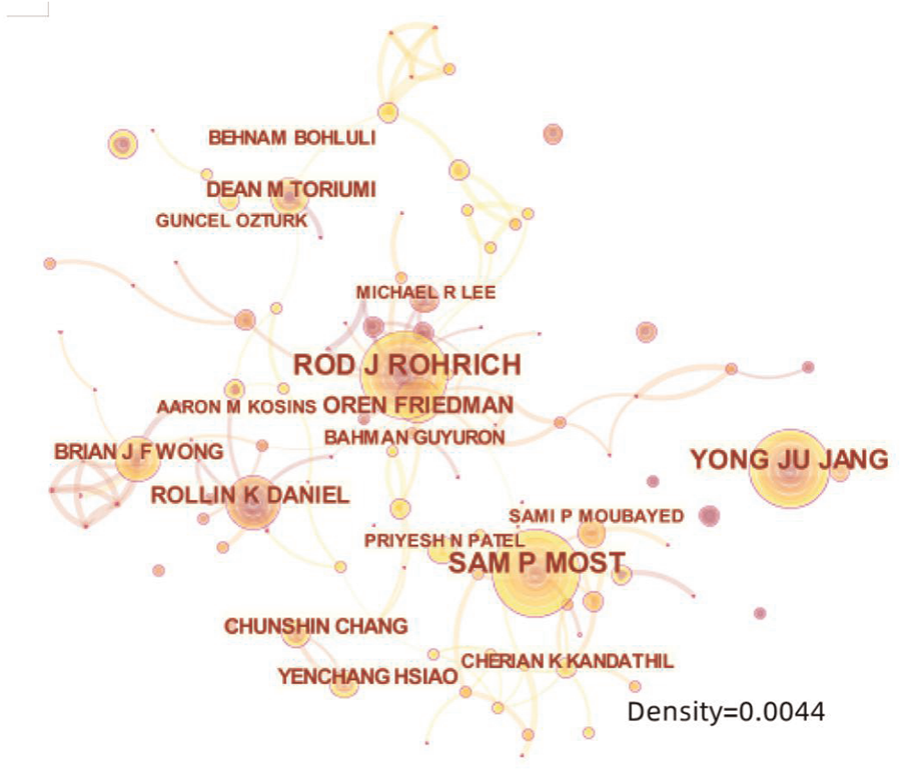
The network map of most productive authors.

**Figure 8:**
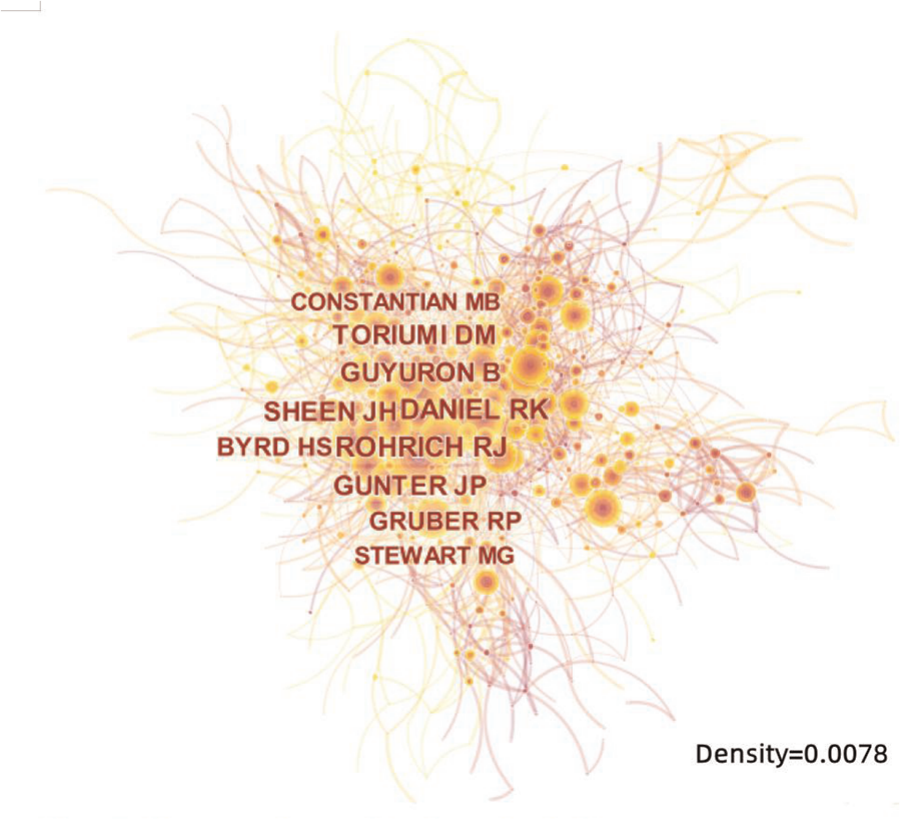
The network map of most co-cited authors.

The 10 authors who published the most literature studies in this study are listed in Table 2. Among which Rohrich RJ, from the Dallas Plastic Surgery Institute, and Division of Plastic Surgery, Baylor College of Medicine, United States, ranked first with 53 literature studies. The second was Most SP from the Department of Otolaryngology-Head and Neck Surgery, Stanford University School of Medicine, United States, with 47 literature studies, followed by Yang YJ from the Department of Otolaryngology, Asan Medical Center, University of Ulsan College of Medicine, Korea, with 41 literature studies. The above three scholars are authorities in RP research who have contributed greatly during the past 10 years. The annual output of articles written by the top 10 authors from 2012 to 2021 is shown in Figure 6, which means that the darker the color is or the larger the circle is, the more articles are produced in that year. Meanwhile, we calculated the citation information for authors, visualizing them in a network by CiteSpace. Rhorich RJ, with 644 literature studies, ranked first in the top 10 co-cited authors, followed by Daniel RK (486), Guyuron B (413), Toriumi DM (406), and Gunter JP (338) (Table 3). The top 10 specialists conducted a huge quantity of research and laid a solid foundation for the development of RP. Also, the centrality of the top 10 authors was more than 0.15, suggesting that they had formed a very influential core scholar group in the domain of rhinoplasty research. Then we use CiteSpace to map the integrated information of authors (Figure 7) and co-cited authors (Figure 8).

**Table 2:** The top 10 productive authors and co-cited authors contributed to publications in rhinoplasty research.

Rank	Author	Article counts	Centrality	Total number of citations	First author counts	First author citation counts	Corresponding author	Corresponding author citation counts	Co-cited author	Citation counts	Centrality
1	Rohrich RJ	53	0.05	475	17	192	34	344	Rohrich RJ	644	0.01
2	Most SP	47	0.01	313	1	11	35	221	Daniel RK	486	0.02
3	Jang YJ	41	0.00	313	9	76	39	299	Guyuron B	413	0.01
4	Daniel RK	23	0.02	494	5	130	10	203	Toriumi DM	406	0.02
5	Friedman O	22	0.03	115	4	20	11	96	Gunter JP	338	0.01
6	Chang CS	19	0.00	51	1	11	2	3	Sheen JH	321	0.01
7	Wong BJF	18	0.01	109	1	1	19	81	Byrd HS	253	0.01
8	Toriumi DM	17	0.02	152	7	70	12	120	Gruber RP	251	0.02
9	Hsiao YC	17	0.00	36	3	10	14	23	Constantian MB	226	0.02
10	Bohluli B	14	0.00	48	10	30	10	19	Stewart MG	186	0.03

**Table 3:** The top 10 most active journals that published articles in rhinoplasty research (sorted by IF).

Rank	Journal title	IF	JCR	Article counts	Percentage	H-index	Total number of citations	Average number of citations
1	*Plastic and Reconstructive Surgery*	4.73	Q1	190	7.34	28	1361	7.16
2	*JAMA Facial Plastic Surgery*	4.611	Q4	123	4.75	21	963	7.83
3	*Aesthetic Surgery Journal*	4.283	Q2	153	5.91	23	989	6.46
4	*Laryngoscope*	3.325	Q3	61	2.36	16	348	5.70
5	*Journal of Plastic Reconstructive and Aesthetic Surgery*	2.74	Q2	61	2.36	12	189	3.10
6	*European Archives of OTO-Rhino- Laryngology*	2.503	Q2	55	2.12	13	288	5.24
7	*Aesthetic Plastic Surgery*	2.326	Q3	253	9.77	18	730	2.89
8	*Facial Plastic Surgery Clinics of North America*	1.918	Q4	84	3.24	16	405	4.82
9	*Facial Plastic Surgery*	1.446	Q3	266	10.27	17	922	3.47
10	*Journal of Craniofacial Surgery*	1.046	Q4	271	10.46	12	355	1.31

### The top 10 ranked journals on publication

From 2012 to 2021, 219 journals have published literature studies in the field of RP research, among which the top 10 most popular journals contributed 1,517 of all 2,590 pieces of literature studies on RP in our study (58.57%) (Table 3). Of these, the top three journals are *Plastic and Reconstructive Surgery, Aesthetic Surgery Journal*, and *JAMA Facial Plastic Surgery,* which accounted for about 18.34% of all the obtained publications. The highest influence factor (IF) belonged to *Plastic and Reconstructive Surgery* (4.73), followed by *JAMA Facial Plastic Surgery* (4.61), *Aesthetic Surgery Journal* (4.28), *Laryngoscope* (3.33), and *Journal of Plastic Reconstructive and Aesthetic Surgery* (2.74). According to the JCR 2021 standards, *Plastic and Reconstructive Surgery* mentioned above is classified as Q1; *Aesthetic Surgery Journal*, *Journal of Plastic Reconstructive and Aesthetic Surgery*, and *European Archives of OTO-Rhino-Laryngology* are classified as Q2; and *Laryngoscope*, *Aesthetic plastic surgery*, *Facial plastic surgery* are classified as Q3; and *JAMA Facial Plastic Surgery*, *Facial Plastic Surgery Clinics of North America*, and *Journal of Craniofacial Surgery* are classified as Q4.

### The top 10 cited literature studies about RP

The details of the top 10 cited articles are listed in Table 4. As for the most-cited literature studies, the literature studies by Mendelson B, Sullivan CD, and Saban Y were ranked first, second, and third, with 143, 118, and 98 citations, respectively. They were published in *Aesthetic Plastic Surgery, JAMA Facial Plastic Surgery*, and *Aesthetic Surgery Journal.* Two of the top 10 authors, Most SP and Daniel RK, conducted the top 10 most-cited papers.

**Table 4:** The top 10 high-cited papers in rhinoplasty research from 2012 to 2021.

Rank	Title	Journal	Corresponding authors	Publication years	Total citations
1	Changes in the facial skeleton with aging: implications and clinical applications in facial rejuvenation	*Aesthetic Plastic Surgery*	Mendelson B	2012	143
2	A systematic review of patient-reported nasal obstruction scores defining normative and symptomatic ranges in surgical patients	*JAMA Facial Plastic Surgery*	Sullivan CD	2014	118
3	Dorsal preservation: the push down technique reassessed	*Aesthetic Surgery Journal*	Saban Y	2018	98
4	Facial feminization surgery: current state of the art	*International Journal of Oral and Maxillofacial Surgery*	Altman K	2012	79
5	The role of piezoelectric instrumentation in rhinoplasty surgery	*Aesthetic Surgery Journal*	Kosins AM	2016	78
6	Printability of pulp derived crystal, fibril and blend nanocellulose-alginate bioinks for extrusion 3D bioprinting	*Biofabrication*	Whitaker IS	2019	77
7	The osseocartilaginous vault of the nose: anatomy and surgical observations	*Aesthetic Surgery Journal*	Daniel RK	2015	77
8	The 10-item standardized cosmesis and health nasal outcomes survey (SCHNOS) for functional and cosmetic rhinoplasty	*JAMA Facial Plastic Surgery*	Most SP	2018	76
9	Complications associated with autologous rib cartilage use in rhinoplasty a meta-analysis	*JAMA Facial Plastic Surgery*	Jin HR	2015	74
10	Antibiotic prophylaxis for preventing surgical-site infection in plastic surgery: an evidence-based consensus conference statement from the American Association Of Plastic Surgeons	*Plastic and Reconstructive Surgery*	Ariyan S	2015	71

### Clustering analysis of the RP hotspots

**Figure 9:**
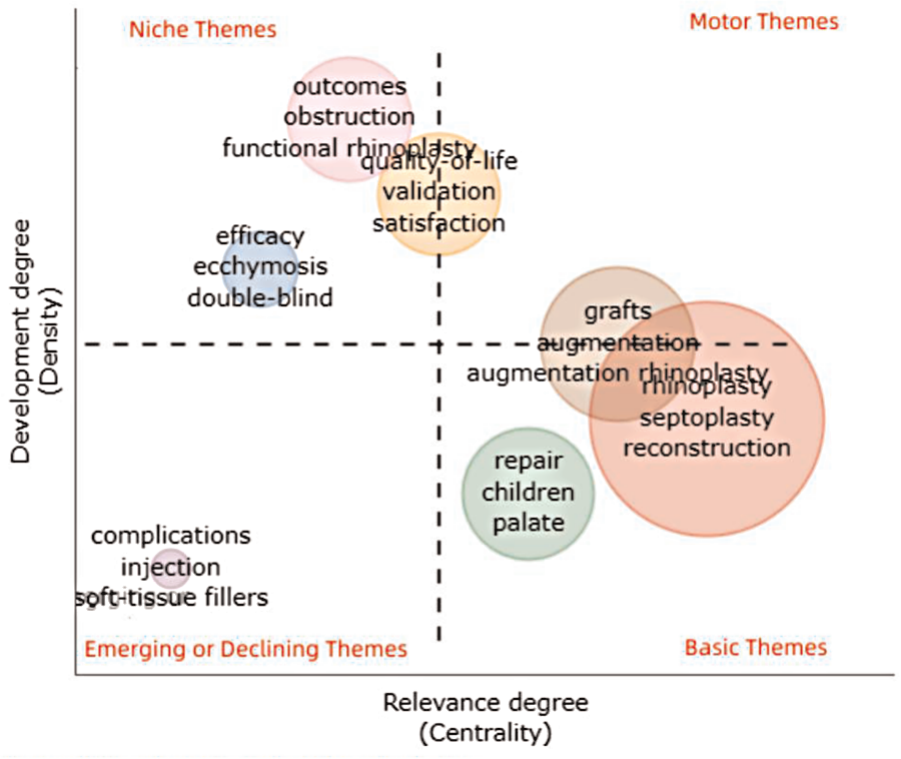
The thematic map of keywords plus.

**Figure 10:**
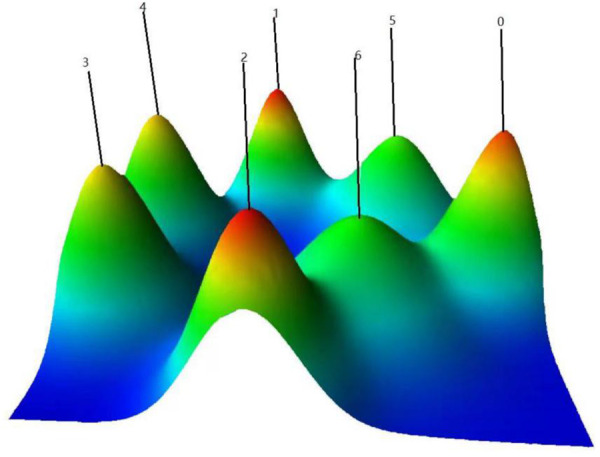
Mountain visualization of biclustering of highly frequent keywords and source literature studies on rhinoplasty research.

**Figure 11:**
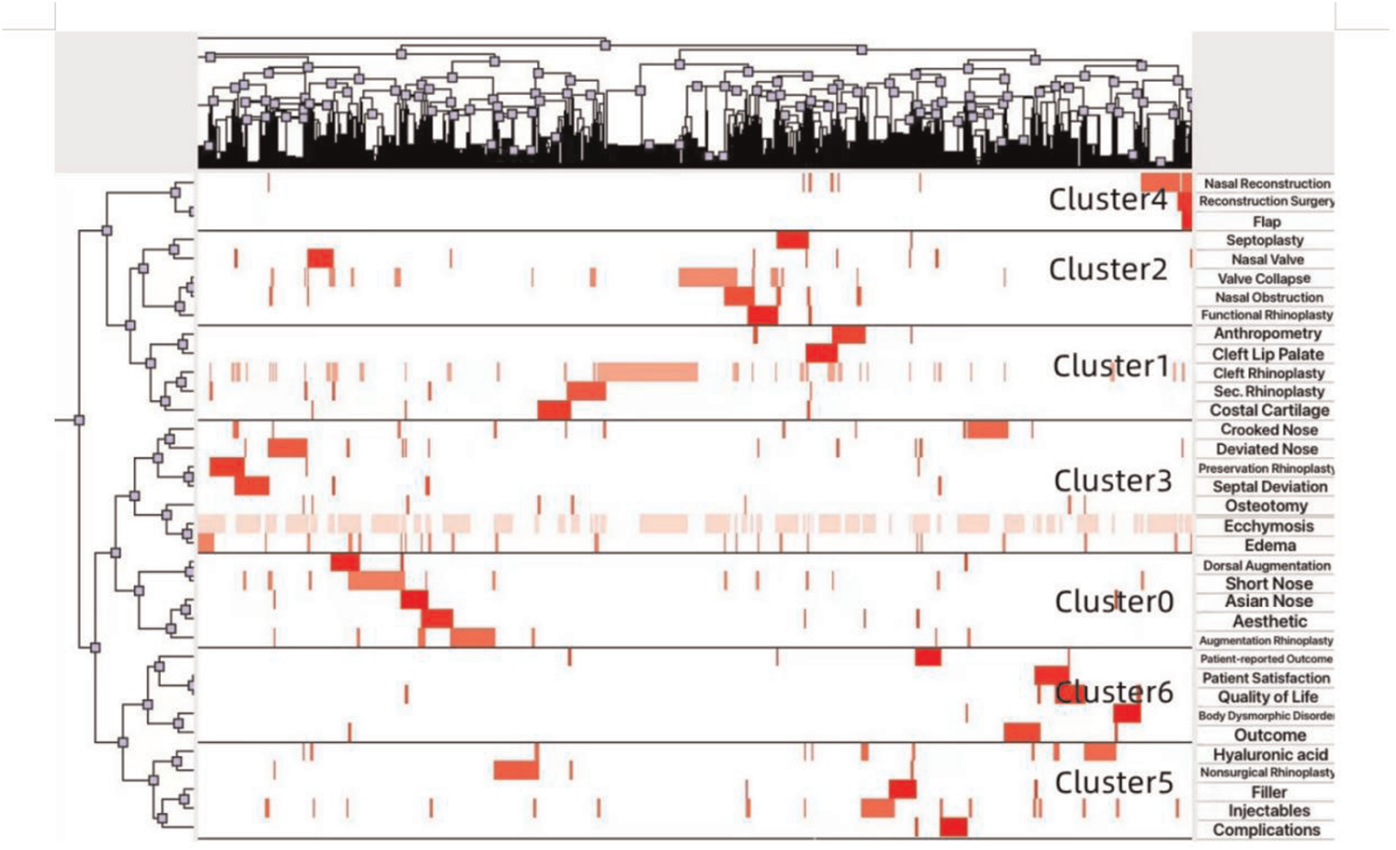
Matrix visualization conducted by gCLUTO (version1.0, University of Minnesota).

Figure 9 illustrates the detailed thematic map in the field of RP. In order to get a more comprehensive view, we screened the keywords with frequency higher than 25 (25 included), which accounted for 28.03% of all words, and used BICOMB combined with gCLUTO to sort out 7 distinctive clusters. Thus, closely connected keywords will be identified and categorized into one cluster and systematical knowledge structure and trends in RP field will be reorganized. Moreover, mountain (Figure 10) and matrix visualizations (Figure 11) are depicted to visualize the correlations between keywords and source literature. The above high-frequency keywords are divided into seven categories, and representative literature studies of each category were deeply studied and further summarized. In the end, we have concluded seven hotspots as follows:

Cluster 0: Augmentation rhinoplasty; Cluster 1: Cleft rhinoplasty; Cluster 2: Functional rhinoplasty; Cluster 3: Osteotomy; Cluster 4: Crooked/deviated nose; Cluster 5: Nonsurgical rhinoplasty; Cluster 6: Outcome and PROMs.

## Discussion

Though the research directions of rhinoplasty are relatively extensive, summary and analysis of relating hotspots are still hard to find. Keywords can represent the research content of literature studies and the order of magnitude keywords can depict the current research status and trend of the region. According to a qualitative and co-word biclustering analysis by bibliometric software, analogical keywords can be recognized and classified into clusters. Paying attention to and digging deeply into these clusters can help identify valuable future directions.

In this article, we get seven clusters through the biclustering analysis. Cluster 0 mainly concentrates on augmentation rhinoplasty. Since many Asians have a short nose that is generally characterized as having low dorsum, short columella, poorly defined nose tip, flare nostril shape, and wide alar base (9), augmentation surgery has become increasingly popular to correct above features for esthetic purposes. In the past, simple augmentation of the dorsum using an implant has been widely operated and reported; with the improvement of esthetic cognition, recent literature studies show that this procedure will result in disharmony of the nasal base, leading to visible nostrils, a longer supratip lobule, and shorter columella (10–12). Accordingly, it is commonly believed that Asians need to augment and elongate the nose simultaneously to achieve satisfying augmentation rhinoplasty (13).

Cluster 1 mainly concentrates on cleft rhinoplasty. Since primary rhinoplasty should be performed at the time of cleft lip repair, it becomes a common practice among cleft surgeons (14). After that, more related publications from 2012 to 2021 focuses on secondary cleft rhinoplasty. Key elements in successfully secondary rhinoplasty are the replacement of bony structures and reconstruction of the absent/asymmetric cartilages. The increasing use of autogenous costal cartilage enables the surgeons to create various different grafts and fortify the soft tissue to resist persistent deformities (15). Other keywords in this cluster deserve attention is anthropometry. Over the years, surgeons have been realizing that it is essential to know how nasolabial features change over time after the primary repair and subsequent surgical and orthodontic interventions, which provides references about residual deformities that may need further revision, and 3D anthropometric technology has been developed rapidly and widely used nowadays in cleft anthropometry (16). Series of longitudinal studies have been implemented according to different interventions (17–20).

Cluster 2 mainly concentrates on functional rhinoplasty. The functional rhinoplasty patient requires improvements in breathing and olfaction instead of shape (21). Internal nasal valve obstruction, external nasal valve collapse, and septal deviation are the three major causes of nasal airway obstruction. Among them, septal deviation is the most common reason and a prevalent problem in the general population (22). Many septoplasty techniques including endonasal, endoscopic, and open procedures have been described. Much of the literature in the latest 10 years focus on comparing the above approaches. However, each of the septoplasty techniques contains advantages and disadvantages, but none of them has been proved as the most successful means to correct septal deviation (23, 24).

Cluster 3 mainly concentrates on crooked/deviated nose and osteotomy. Both extrinsic forces and intrinsic forces can lead to nasal structure distortion and nasal deviation; nevertheless, majority of them share septal deviation in common. Key operative principles for correcting the crooked nose include rectification of deviated septum and nasal osteotomy. Generally, hybridization of preservation and structural rhinoplasty is required (25, 26). Due to the emergence of potential negative implications of excision techniques, surgeons nowadays pay much interest to dorsal preservation. The maintenance of the structural integrity at the nasal keystone, dorsal esthetic lines, and the patency of the internal nasal valve are points of dorsal preservation (27, 28). In addition, osteotomy is closely associated with postoperative eyelid edema and ecchymosis. Though much publications report above complications in different surgical methods, there is no consensus on which method is the most effective in reducing them (29).

Cluster 4 mainly concentrates on nasal reconstruction and reconstruction surgery. The missing tissue of nasal is categorized as lining, support, and cover, corresponding to mucosa, cartilaginous and bony skeleton, and the overlying skin (30). Literature studies about reconstruction published from 2012 to 2021 still concentrate on the above three aspects, especially on the flaps. Apart from the cases of classical flaps, many newly flaps such as the prelaminated temporoparietal osteofascial flap (31) and modified flaps such as the nasomentolabial flap (32) are reported.

Cluster 5 mainly concentrates on nonsurgical rhinoplasty (NSR). Comparing to the golden standard—surgical rhinoplasty for nasal correction—NSR possesses the advantages including lower cost, less downtime, and immediate effect, and thus are more and more favored by modern people (33). Also, NSR is more easily carried out by a wider range of practitioners and has a more mild learning curve than RP (34). The most well-known types of fillers include hyaluronic acid (HA), collagen, paraffin, and liquid silicon (35). Experienced doctors report their unique injection methods (36–38) without reaching a consensus. Typically, injection rhinoplasty is fairly safe but complications may occur occasionally. So many surgeons will introduce the technique they use during the procedure to avoid the complications. However, serious complications such as dermal necrosis due to vascular obstruction are rare but still reported (39).

Cluster 6 mainly concentrates on outcome and patient satisfaction of the rhinoplasty. In recent years, there is an increasing trend to use health-related quality of life (QoL) questionnaires or multiple patient-reported outcome measures (PROMs) to assess patient satisfaction after surgical procedure. An ideal assessment of RP outcome should have objective means of evaluation covering shape, function, and psychology (40). Currently available and most widely used outcome measures in rhinoplasty include Rhinoplasty Outcomes Evaluation (ROE), The Nasal Obstruction Symptom Evaluation (NOSE), FROI-17, Rhinoplasty Health Inventory and Nasal Outcomes Scale Description (RHINO), FACE-Q, and Standardized Cosmesis and Health Nasal Outcomes Survey Description (SCHNOS). Those specialized rhinoplasty PROMs can provide tremendous feedback information to surgeons; thus, the related research studies flourished in the last 10 years. In addition, the Body Dysmorphic Disorder (BDD) Questionnaire has also become essential to surgeons for assessing patients before surgery. It can reveal the psychological state of the patient and help understand the demands of patient, serving as an effective way to avoid the outcome of dissatisfied patients and depressed doctors (41).

Though we have analyzed the publications on RP from 2012 to 2021 as comprehensively as possible, some limitations still exist. First, since the RP databases update incessantly from 1870 to date, we only selected the publications from 2012 to 2021. Therefore, a discrepancy may exist between this bibliometric analysis and real publication conditions. In addition, the amount of RP literature may increase rapidly with the breakthrough of treatment methods or novel concept. Take Turkish delight, for example, a few relative literature studies were published between 2000 and 2011; however, 19 articles about Turkish delight were published in 2011–2021 and thus making it a highly frequent major keyword. Since it lacks the tight and continuous correlation with other conventional keywords, the biclustering method did not classify it into any prominent cluster. Most of the published rhinoplasty series are the final works of very prestigious experts working in a stationary location for decades, which limit the chances of collaborative work of different institutions and are also reflected in the calculated centrality and density. The majority of the augmentation RPs are from East like Korean and China, while most osteotomies that aim at hump nose are from NA and Europe. Surgical techniques and research trends can vary differently among regions.

## Conclusions

We conducted a comprehensive summary of the publication information of RP-related literature studies in the latest 10 years from 2012 to 2021, pointing out the research trends over time. In general, the purpose of RP is mainly divided into two aspects: the appearance (including esthetic deficiency and deformity) and the function of nose. The literature studies about esthetic RP mainly focus on augmentation RP, which are usually reported by Asians. As for deformity, it mainly concentrates on cleft RP and nasal defect. 3D anthropometry has been used more popular than ever for cleft rhinoplasty, but systematic and large-scale measurement data have not been reported yet, which deserves multiple medical centers to cooperate in the future. The nasal defect mainly involves nasal reconstruction. Both classic flaps and innovative flaps have been reported incessantly; however, existing surgical methods still could not meet the needs of special types of patients or patients with higher requirements for appearance. More suitable ways need to be explored. For the function of nose, nasal obstruction that resulted from septal deviation accounted for over 90% of all related literature studies. The three mainstream surgical techniques including endonasal, endoscopic, and open procedures have been elaborated. However, the comprehensive advantages and drawbacks about the above three still need further studies. Another hotspot deserves scholars’ attention lies in the valuation of patients’ outcome. The patient self-reported outcome evaluation scale has become a very popular evaluation method. However, the scale is scored by the patients themselves, which has subjective consciousness defects. How to evaluate the postoperative outcome more reasonably, objectively, and comprehensively is a problem that doctors need to think about carefully.

## Data Availability

The raw data supporting the conclusions of this article will be made available by the authors, without undue reservation.
